# Study on the mechanism of Wnt/β-catenin pathway mediated by pterostilbene to reduce cerebral ischemia-reperfusion injury

**DOI:** 10.17305/bb.2025.11743

**Published:** 2025-02-17

**Authors:** Yang Jin, Chunwang Fu, Ming Guo, Qiang Yang

**Affiliations:** 1 Shenyang Xingqi Pharmaceutical Co., Ltd., Shenyang, China

**Keywords:** Pterostilbene, PTE, cerebral ischemia-reperfusion injury, CIRI, ferroptosis, Wnt/β-catenin pathway

## Abstract

Cerebral ischemia-reperfusion injury (CIRI) is the primary cause of damage following ischemic stroke, with ferroptosis serving as a key pathophysiological factor in CIRI. Pterostilbene (PTE) has been shown to reduce cerebral ischemic injury, but whether its mechanism of action involves ferroptosis remains unclear. In this study, an in vitro model of mouse hippocampal neuron (HT22) cell injury and an in vivo mouse CIRI model were established. Treatments included PTE, the ferroptosis activator Erastin, and the Wnt signaling pathway inhibitor (Dkk-1). Cell damage was assessed using flow cytometry, MTT assay, lactate dehydrogenase (LDH) release assay, and Calcein-AM/PI staining. Oxidative stress and ferroptosis in cells and tissues were evaluated using biochemical kits and fluorescence staining. Additionally, histopathological staining was performed to assess brain tissue damage, while qRT-PCR and Western blot analyses were used to measure ferroptosis-related factors and Wnt/β-catenin pathway-related proteins in both cells and tissues. HT22 cells subjected to injury exhibited decreased viability and increased cell death (*P* < 0.05). Similarly, CIRI mice demonstrated pronounced cerebral infarction and neuronal damage. Ferroptosis, characterized by elevated levels of iron ions, lipid peroxides (ROS and MDA), and reduced antioxidant enzymes (GSH and GPX4), was significantly increased in both cells and tissues (*P* < 0.05). Correspondingly, ferroptosis-related protein levels were elevated (*P* < 0.05), while Wnt/β-catenin pathway-related protein levels were significantly decreased (*P* < 0.05). Treatment with Erastin and Dkk-1 exacerbated neuronal damage, intensified ferroptosis, and inhibited the Wnt/β-catenin pathway. Conversely, PTE treatment activated the Wnt/β-catenin pathway, reduced ferroptosis, and improved neuronal damage. Specifically, PTE upregulated the Wnt/β-catenin pathway, decreased peroxide accumulation, and antagonized ferroptosis, ultimately mitigating CIRI. These findings suggest that PTE protects against CIRI by modulating the Wnt/β-catenin pathway and alleviating ferroptosis-induced damage.

## Introduction

Ischemic stroke is a significant illness for threaten human health, and its pathogenesis is mainly ischemic hypoxic lesions after blockage of important intracranial blood vessels [1]. The occurrence of ischemic hypoxic lesions in brain tissue can cause irreversible ischemic necrosis of brain tissue, leading to severe neurological dysfunction and even death [2]. Cerebral ischemia-reperfusion injury (CIRI) is the most important pathological injury after ischemic stroke [3], and it is also the main direction of current basic experimental and clinical trial research. CIRI refers to the brain tissue after a period of ischemia, re-supply of blood, the blood supply area of the organization to produce a large number of active substances caused by brain tissue damage, patients may have memory, language, sports, and other aspects of the disorder [4]. CIRI is a complicated biological mechanism involving many factors, which is considered to be associated with inflammatory response, apoptosis, and oxidative stress [5, 6]. The length and intensity of ischemic damage determine the recoverability of the injury response and the ultimate survival rate of the tissue [7]. Therefore, effective treatment is particularly important for CIRI. Unfortunately, there is currently no specific clinical treatment [8, 9]. It is required to examine the process of CIRI and find new prevention and treatment methods.

Ferroptosis is a kind of regulatory cell death. After brain injury, the deposition of Fe^2+^ in the damaged area causes elevated oxidative stress and inflammatory response, increased apoptosis, etc., resulting in neuronal death and neurological dysfunction [10]. Ferroptosis is a biological process controlled by various genes, primarily related to iron balance and lipid peroxidation metabolism [11–13]. The features of ferroptosis are characterized by rupture of plasma membrane, mitochondrial atrophy, normal nucleus size, swelling of organelles and formation of vesicles, and non-aggregation of chromatin. Its biochemical characteristics are mainly manifested as the inhibition of cystine/glutamate transport system, the buildup of reactive oxygen species (ROS) caused by intracellular iron overload and the increase of lipid peroxidation [14]. At present, it is believed that various physiological processes, such as neurodegeneration and ischemic organ damage may be related to ferroptosis [15–17]. Studies have shown that iron buildup was noted in CIRI rats [18], and ferroptosis inhibitor treatment can diminish cerebral infarction and enhance the behavioral defects of CIRI rats [19]. Astragaloside IV inhibits ferroptosis by affecting the key proteins of ferroptosis and anti-lipid peroxidation, thereby improving the sensory and motor dysfunction of middle cerebral artery occlusion (MCAO) rat models [20, 21]. Vitexin can reduce the volume of brain injury in MCAO rat model, reduce histopathological damage and improve mitochondrial function, reduce ROS production, and play a protective role in ferroptosis [22]. Therefore, suppression of ferroptosis is a novel approach for treating CIRI [23–25].

Pterostilbene (PTE, 3,5-dimethoxy-4′-hydroxystyrene), is a resveratrol-like derivative with high lipid solubility and bioavailability. It has many pharmacological effects, such as antioxidant, anti-inflammatory, etc. [26–28], and the blood–brain barrier permeability is also high [29]. PTE has a clear inhibitory effect on tumor factors, inflammatory factors, apoptosis, and anti-oxidative stress in many diseases [30, 31]. Studies have shown that PTE is closely related to ferroptosis. PTE improves oxidative damage and ferroptosis of human ovarian granulosa cells by regulating Nrf2/HO-1 pathway [32], and also reduces heart failure by inhibiting myocardial ferroptosis [33]. At the same time, it also has the exact effect of inhibiting apoptosis and anti-inflammation in nervous system diseases, and even the mechanism in cerebral injury has been initially verified. PTE can exert neuroprotective and anti-inflammatory effects on MCAO by inhibiting cyclooxygenase-2 (COX-2) [34], and can also improve neurological dysfunction and neuroinflammation after ischemic stroke through HDAC3/Nrf1-mediated microglia activation [35]. The neuroprotective impact of PTE is associated with inflammation in microglia [36]. However, it is unknown whether PTE can improve CIRI by inhibiting ferroptosis.

Therefore, we hypothesize that PTE can improve CIRI by inhibiting ferroptosis. Based on this hypothesis, this study intends to construct a HT22 cell injury model by oxygen-glucose deprivation/re-oxygenation (OGD/R). At the same time, a mouse CIRI model was constructed by transient MCAO to investigate the effects of PTE on cell injury, iron homeostasis and lipid peroxidation metabolism in order to elucidate the impacts of PTE on CIRI and its specific process, and provide reference for clinical treatment of CIRI with PTE.

## Materials and methods

### Cell grouping and processing

Mouse hippocampal neuron cells HT22 were procured from the Cell Bank of Chinese Academy of Sciences (GNM47, Shanghai, China) and cultured in DMEM medium containing 10% fetal bovine serum (C0235, Beyotime, Shanghai, China) at 37 ^∘^C and 5% CO_2_.

For the construction of OGD/R model, cells in logarithmic growth phase were collected and inoculated in glucose-free DMEM medium, and then cultured in an ischemic chamber containing 95% CO_2_ for 2 h (hypoxia). Then the OGD/R group was exchanged for normal medium and continued to be cultured for 24 h (reoxygenation). Intervention was performed 1 h before modeling, the OGD/R + 2.5 µM PTE group was given 2.5 µM PTE, the OGD/R + 5 µM PTE group was given 5 µM PTE, the OGD/R + 10 µM PTE group was given 10 µM PTE, the OGD/R + Erastin group was given 10 µM ferroptosis activator Erastin (S7242, Selleck, Shanghai, China), the OGD/R + 10 µM PTE + Erastin group was given 10 µM PTE and 10 µM Erastin, the OGD/R + Dkk-1 group was given 20 µg/mL Wnt signaling pathway inhibitor Dkk-1 (A2522, Selleck), the OGD/R + 10 µM PTE + Dkk-1 group was given 10 µM PTE and 20 µg/mL Dkk-1. The control group was not treated, and the 2.5, 5, and 10 µM PTE groups were cultured normally.

### MTT assay

The HT22 cell suspension was inoculated in 96-well plates at a density of 1 × 10^4^ cells/well. After modeling and administering, 20 µL MTT solution (M8180, Solarbio, Beijing, China) was added and incubated for 4 h. After removing the supernatant, 200 µL DMSO solution (D8371, Solarbio) was introduced and placed on a shaker for 10 min. Then the OD value at 570 nm was tested using a microplate reader to calculate cell viability.

### Lactate dehydrogenase (LDH) assay

The cell damage was tested by LDH cytotoxicity assay kit (C0016, Beyotime, Shanghai, China). After modeling and administering HT22 cells, in addition to the eight groups of OGD/R, there were also cell-free culture medium holes (blank control holes) and untreated control holes for measuring the maximum enzyme activity of the sample. One hour before the end of reoxygenation, 20 µL LDH was added to the untreated control hole and mixed, and then continued to be cultured until the end of OGD/R. After centrifugation, 120 µL of supernatant was obtained. The OD value at 490 nm was tested by the microplate reader. The experimental results are shown as the percentage compared with the cell blank treatment group.

### Calcein-AM/PI staining

The live/dead cells was tested by Calcein-AM/PI staining kit (CA1630, Solarbio). After HT22 cell modeling and administration, the cells were digested with trypsin, washed with Assay Buffer and adjusted to 1 × 10^5^ cells/mL. Calcein-AM (10 µL) was added and incubated in dark for 20 min. 30 µL PI stock solution was introduced and stained in dark for 5 min; the staining solution was discarded, the cells were resuspended after PBS cleaning, and 3 µL was dropped on the slide. Then, the 490 nm excitation filter was used under the fluorescence microscope to observe the image.

### Flow cytometry

About 1 × 10^6^ cells were obtained and washed with PBS, 500 µL binding buffer was added and gently suspended. Then 10 µL PI were mixed and reacted in dark for 15 min. The samples were transferred to a flow-specific loading tube, and cell death was tested by flow cytometry within 1 h and the death rate was calculated.

### DHE staining was used to test intracellular superoxide anion

After modeling and administration, the cells was calibrated to 1 × 10^6^ cells/mL, 500 µL of DHE (S0063, Beyotime) with a concentration of 10 µM was supplemented, cultured for 20 min. The cells were examined and captured under the fluorescence microscope using blue or green light excitation.

### MitoSox staining was used to test superoxide anion levels in mitochondria

MitoSOX Red (HY-D1055, MCE, Shanghai, China) was diluted with anhydrous DMSO to prepare a 5 mM stock solution, and then diluted again with a preheated PBS solution to get a 5 µM MitoSOX Red working solution. HT22 cell was grown on sterile coverslips. After removal, 100 µL MitoSOX Red working solution was added and incubated for 20 min. Then the cell was washed with medium and observed under a fluorescence microscope.

### Immunofluorescence

HT22 cells were fixed in acetone and washed with PBS. The cells were permeabilized in TBS solution containing 0.25% TritonX-100 for 10 min, and blocked for 1 h. Glutathione peroxidase 4 (GPX4) antibody (ab125066, 1: 50, Abcam) was incubated overnight. PBS was rinsed three times, the second antibody goat anti mouse IgG (GB25301, 1:5000, Servicebio) was incubated for 1 h, the tablet (S2110, Solarbio) was sealed, and the fluorescence microscope was observed and photographed.

### Animal grouping and processing

All mice were arbitrarily split into seven groups (eight mice/group): sham operation (Sham) group, cerebral ischemia-reperfusion (CIR) (MCAO) group, MCAO + 2.5 mg/kg PTE (MCAO + 2.5 mg/kg PTE) group, MCAO + 5 mg/kg PTE (MCAO + 5 mg/kg PTE) group, MCAO + 10 mg/kg PTE (MCAO + 10 mg/kg PTE) group, MCAO + 20 µg/mL Dkk-1 (MCAO + Dkk-1) group. MCAO + 10 mg/kg PTE + 20 µg/mL Dkk-1 (MCAO + 10 mg/kg PTE + Dkk-1) group. The mice were anesthetized using an intraperitoneal injection of 2% pentobarbital at a dosage of 80 mg/kg, and then the skin was cut along the middle of the neck to separate the subcutaneous tissue. The division of the right common carotid artery was exposed, and the length from the division to the middle cerebral artery was 10–12 mm. The uncoated 6–0 monofilament nylon stitch (tip diameter 0.20 ± 0.01 mm) was placed from the division to obstruct the middle cerebral artery opening. After 1 h of ischemia, the suture was removed and the blood perfusion was restored for 24 h. Sham group did not insert sutures, and other operations were the same. One hour after modeling, mice in PTE group were intraperitoneally injected with 2.5, 5 and 10 mg/kg PTE, respectively. MCAO + Dkk-1 group was intraperitoneally injected with 20 µg/mL Dkk-1; the MCAO + 10 mg/kg PTE + Dkk-1 group was intraperitoneally injected with 20 µg/mL Dkk-1 and 10 mg/kg PTE. After 24 h, the neurological score was performed, and the mice were sacrificed to obtain brain tissue.

### Neurological score

The MCAO model was evaluated by Longa scoring method [37], which was divided into five points: 0 was divided into no neurological deficit; one was that the forelimb of the ischemic side cannot stretch when the tail is lifted; two was divided into spontaneous walking to the opposite side of the circle; three was inclined to the opposite side and unable to stand when walking spontaneously; and four was divided into inability to walk spontaneously and loss of consciousness.

### Evans blue leakage

2% Evans blue (3 mL/kg, IE0280, Solarbio) was injected into the tail vein 3 h before dissection. After 1 h, the brain was decapitated, cut into pieces, placed in 50% trichloroacetic acid, and uniformly broken. After centrifugation, 50 µL supernatant was taken and diluted with anhydrous ethanol four times. The OD value at 620 nm was tested by the microplate reader, and Evans blue concentration was calculated.

### Brain wet/dry weight ratio

After anesthesia, the mice were quickly decapitated, and the wet weight of brain tissue was immediately weighed and recorded. Next, the tissue was set in an oven at 105 ^∘^C for 24 h, and weighed repeatedly until constant weight was recorded as dry weight. Brain water content ═ (wet weight-dry weight) / wet weight × 100 %.

### HE staining

The brain tissue was fixed with 4% paraformaldehyde, embedded in paraffin, and then the tissue samples were sliced into 4 µm thick sections. The sections were stained with hematoxylin (C0107, Beyotime) for 15 min, differentiated in 1% acidic alcohol (containing 70% hydrochloric acid) for 30 s, and then stained with 0.5% eosin (G1100, Solarbio) for 3 min. Then the sections were mounted with neutral gum (G8590, Solarbio). The hippocampal CA1 region was observed under a microscope.

### TTC staining

The sections were immersed in 2% TTC staining solution (G3005, Solarbio), stained in dark for 30 min, and fixed in 4% paraformaldehyde for 24 h. White is the cerebral infarction area, and red is the normal brain tissue. The cerebral infarction area in mice was determined with Image J.

### Nissl staining

Nissl bodies were stained by Nissl staining kit (G1434, Solarbio). The sections of brain tissue were stained with methylene blue for 10 min, and placed in differentiation medium for 1 min, treatment with ammonium molybdate solution for 3 min, washing with distilled water to prevent decolorization, and mounting with neutral gum (G8590, Solarbio). The number and morphological changes of Nissl bodies were observed under microscope.

### TUNEL staining

The apoptosis in brain tissue was tested by Tunel kit (C1091, Beyotime). The sections were dewaxed with xylene and hydrated with gradient ethanol. Protease K solution without DNase (20 µg/mL) was added for 30 min. Apoptotic cells were labeled with 50 µL Tunel solution and incubated in dark for 60 min. DAPI staining solution was employed to stain the nucleus for 5 min. The sections were sealed by anti-fluorescence quenching solution, and the positive situation of Tunel was observed by fluorescence microscope.

### Determination of ROS level

ROS was detected using a ROS detection kit (CA1410, Solarbio). HT22 cells were collected, and the brain tissue was homogenized. The supernatant was gathered after centrifugation, then the cells were adjusted to 1 × 10^6^/mL. DCFH-DA was added to a final concentration of 1 µmol/L, incubated for 30 min, washed with PBS, and stimulated with ROS positive control for 30 min. The levels of ROS were tested by flow cytometry.

### Determination of antioxidant enzyme levels

The supernatant of cells and mouse brain tissue was collected. Malondialdehyde (MDA) assay kit and glutathione peroxidase (GSH-px) assay kit (A003-1-2, A005-1-2, Jiancheng Bioengineering Institute) were used to determine MDA and SOD content. The OD value was read at 450 nm on the microplate reader, and the enzyme content was calculated.

### Determination of Fe^2+^ level

Fe^2+^ levels were tested by ferrous ion content detection kit (BC5415, Solarbio). The HT22 cells after different treatments were collected, and the mouse brain tissue was homogenized. After the supernatant was gathered, 200 µL supernatant, standard solution and reagent 1 were taken in different centrifuge tubes, and 100 µL of reagent 2 was added and mixed well. After standing for 10 min, 100 µL of chloroform was supplemented and vortexed for 5 min. After centrifugation, 200 µL of the upper solution was taken. The OD value at 593 nm was tested by microplate reader, and the Fe^2+^ content was calculated.

### qRT-PCR

RNA was abstracted by TransZol Up (ET111-01-V2, TRANS, Beijing, China), and then AMV reverse transcriptase (2621, TAKARA, Tokyo, Japan) was supplied for reverse transcription to obtain cDNA. Then TB Green FAST qPCR (CN830S, TAKARA) was used for PCR reaction. The relative level of mRNA was computed by 2^−ΔΔCt^ method. GAPDH can serve as a control.

The primer sequences: GPX4: F: 5′-CCTCTGCTGCAAGAGCCTCCC-3′; R: 5′-CTTATCCAGGCAGACCATGTGC-3′; FTH1: F: 5′-GCCGAGAAACTGATGAAGCTGC-3′; R: 5′-GCACACTCCATTGCATTCAGCC-3′; ACSL4: F: 5′-CCTTTGGCTCATGTGCTGGAAC-3′; R: 5′-GCCATAAGTGTGGGTTTCAGTAC-3′; COX-2: F: 5′-GCGACATACTCAAGCAGGAGCA-3′; R: 5′-AGTGGTAACCGCTCAGGTGTTG-3′; GAPDH: F: 5′-TGGATTTGGACGCATTGGTC-3′; R: 5′-TTTGCACTGGTACGTGTTGAT-3′.

### Western blot

After the cells and brain tissue were collected, the samples were fully lysed by RIPA lysis buffer. The protein concentration was tested using BCA kit (PC0020, Solebold). All samples were detached by electrophoresis, and the protein was then transferred to the PVDF membranes (YA1700, Solarbio). The membranes were incubated with 5% skimmed milk powder (LP0033B, Solarbio) for 2 h, and GPX4 (ab125066, 1:2000, Abcam), Ferritin heavy chain 1 (FTH1, ab183781, 1:1000, Abcam), Acyl-CoA synthetase long-chain family member 4 (ACSL4, ab155282, 1:10000, Abcam), COX-2 (ab179800, 1:2000, Abcam), β-catenin (ab32572, 1:5000, Abcam), Glycogen synthase kinase 3β (GSK3β, ab93926, 1:1000, Abcam), p-GSK3β (ab68476, 1:1000, Abcam), Wnt3a (ab219412, 1:1000, Abcam) and GAPDH (TA-08, 1:1000, ZSGB-BIO, Beijing, China) incubated them at 4 ^∘^C for one night. On the next day, the membranes were washed with TBST buffer (T1082, Solarbio) and incubated with secondary antibody (1:20000) for 1 h. After five times washed with TBST buffer, ECL (PE0010, Solarbio) reagent was used to react for 2–3 min, and then automatic chemiluminescence imaging system was used for imaging.

### Ethical statement

Fifty-six SPF male C57/BL6 mice, six weeks of age, weighing (20 ± 2) g, provided by Sberfo Biotechnology Co., Ltd. (Beijing, China). Mice were fed under normal conditions (temperature 20–24 ^∘^C, relative humidity 50%–70%, light and dark 12 h/12 h). The pain of experimental animals should be minimized during the experiment. This study was approved by the Experimental Animal Welfare and Ethics Committee of China Medical University (IACUC No. CMUXN2023057).

### Statistical analysis

Each experiment was repeated at least three times, and all data were expressed as mean ± standard deviation. Statistical analysis and image drawing were performed using Graphpad 9.0. One-way ANOVA analysis was used to compare multiple groups. When the results are significant (*P* < 0.05), the Tukey method is used for post-test. *P* < 0.05 was considered statistically significant.

## Results

### PTE reduces OGD/R-induced injury in HT22 cells

**Figure 1.**
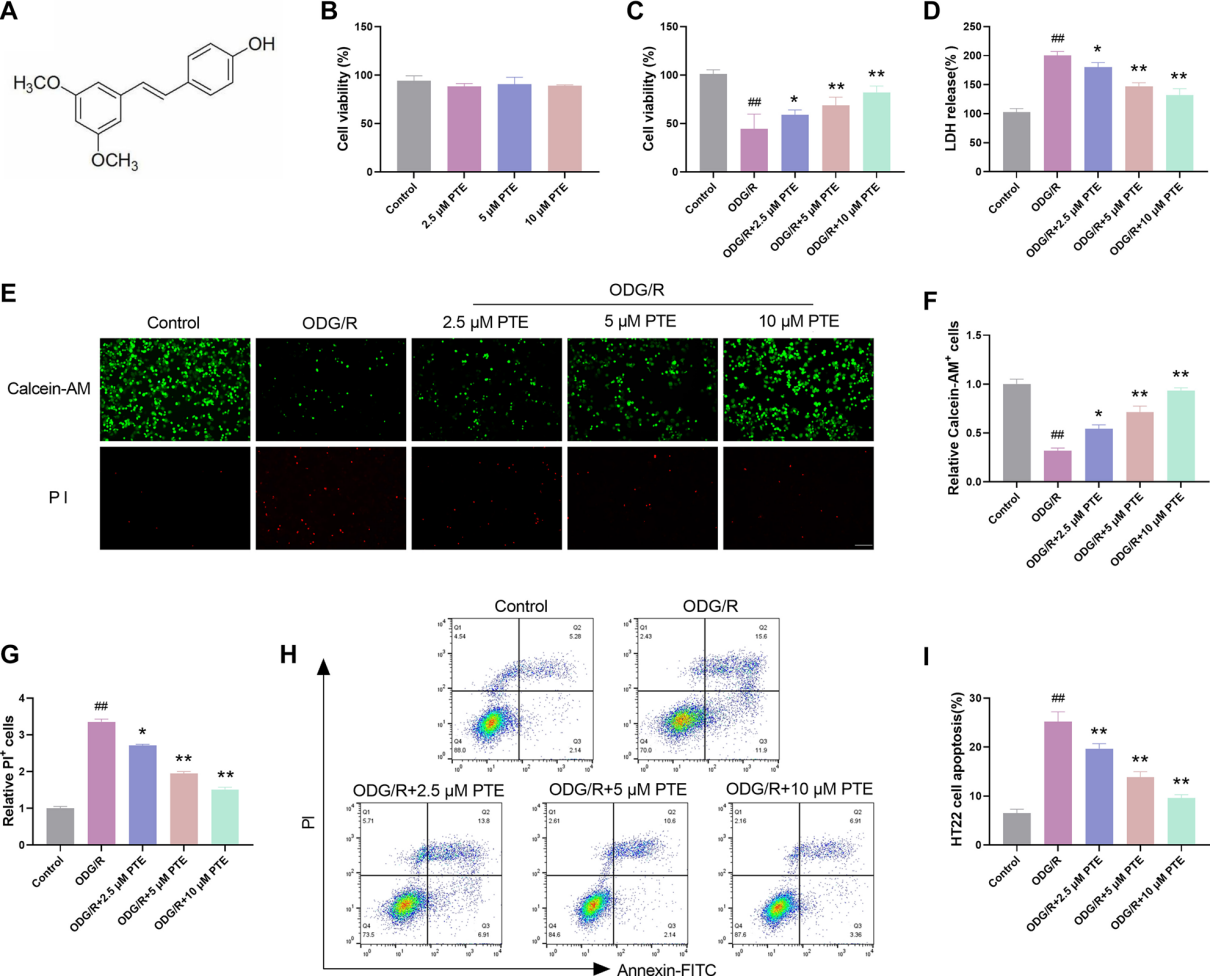
**PTE reduces OGD/R-induced injury in HT22 cells.** (A) PTE structural formula; (B) The cell survival rate under the action of PTE was tested by MTT assay. The PTE dose used in the PTE experiment had no significant cytotoxicity to HT22 cells; (C) The viability of HT22 cells after OGD/R injury was tested by MTT assay, and PTE markedly increased cell viability; (D) The damage degree of HT22 cells treated with different treatments was tested by LDH, and PTE was found to significantly reduce the degree of cell damage; (E–G) Calcein-AM/PI detection of live/dead cells showed that PTE obviously elevated live cell count and declined dead cell count; (H and I) Flow cytometry showed that PTE significantly reduced HT22 cell death rate. Compared with the Control group, ^##^*P* < 0.01; compared with OGD/R group, ^*^P < 0.05, **P < 0.01. PTE: Pterostilbene; OGD/R: Oxygen-glucose deprivation/re-oxygenation; LDH: Lactate dehydrogenase.

The molecular formula of PTE is C_16_H_16_O_3_, and the specific structural formula is displayed in Figure 1A. To confirm whether 2.5 µM, 5 µM, 10 µM PTE has toxic effects on HT22 cells, we first used MTT to detect the cell survival rate under different concentrations of PTE. The selected cells had no substantial impact on cell viability, suggesting that it was a safe concentration (Figure 1B). The viability of HT22 cells expanded with the expand of PTE concentration, indicating the improvement effect of PTE on cell injury (Figure 1C). The release of LDH is regarded as a key sign of cell membrane integrity and is extensively used in cytotoxicity detection. The LDH activity was notably elevated after OGD/R induction, but after treatment with PTE, the LDH activity increased significantly with the increase of PTE concentration (Figure 1D), further indicating that PTE can reduce the degree of OGD/R-induced cell injury. Next, we detected cell viability and death by Calcein-AM/PI staining and flow cytometry. Calcein-AM-labeled living cells increased notably with the expand of PTE concentration, while PI-labeled dead cells showed the opposite trend (Figure 1E–1G), HT22 cell death rate also reduced notably (Figure 1H and 1I). In conclusion, PTE can reduce cell damage by enhancing cell viability under OGD/R injury and reducing cell death.

### PTE reduces OGD/R-induced oxidative stress

**Figure 2.**
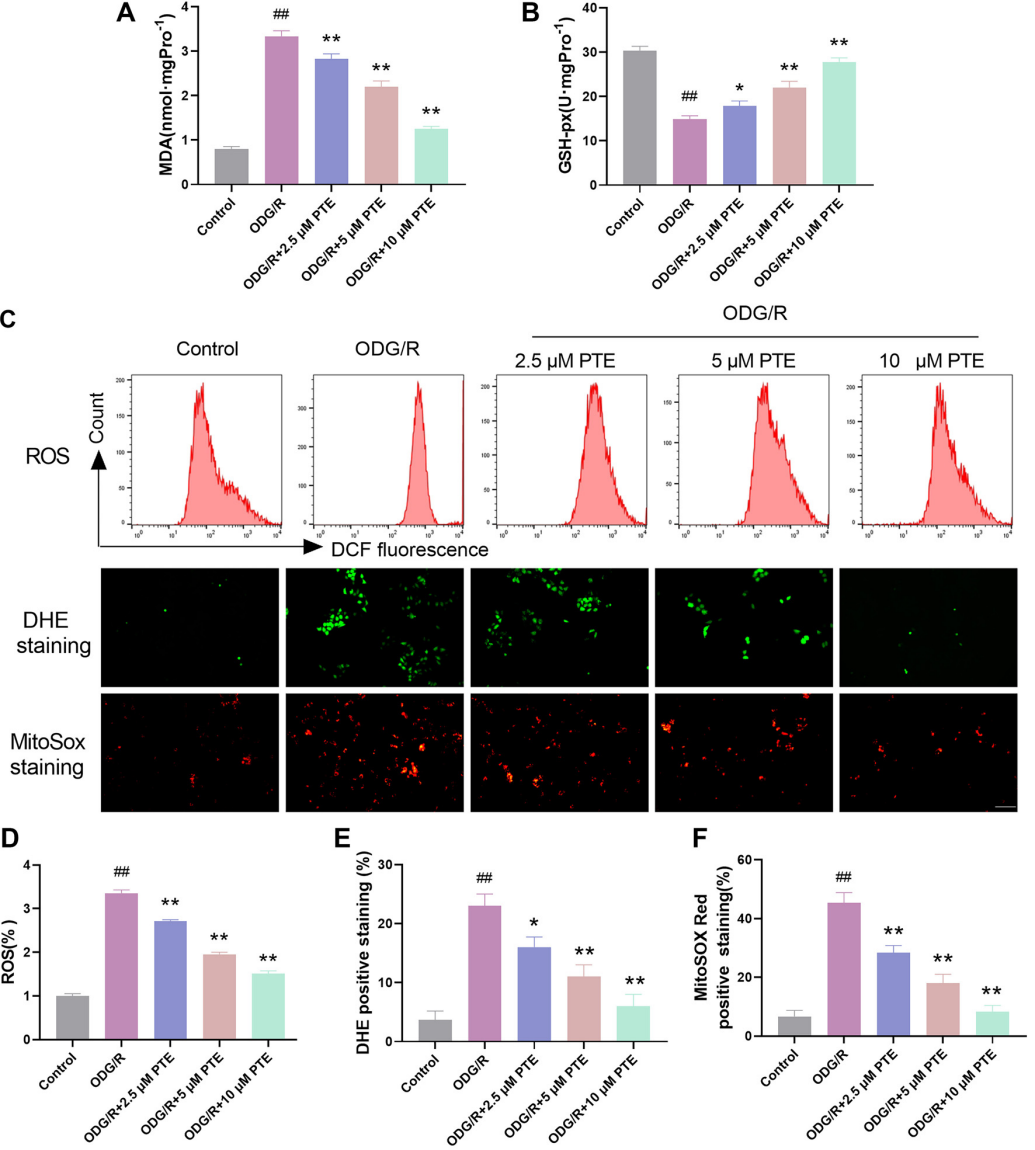
**PTE reduces OGD/R-induced oxidative stress.** (A) The MDA content was detected by the kit, and PTE obviously reduced OGD/R-induced increase in MDA content; (B) The GSH-Px content was detected by kit, which showed that PTE obviously elevated the content of GSH-Px; (C–F) The levels of ROS, superoxide anion and mitochondrial ROS were detected by flow cytometry, DHE and MitoSox, respectively. They were significantly increased after OGD/R induction, and decreased significantly after PTE treatment. Compared with the Control group, ^##^*P* < 0.01; compared with OGD/R group, ^*^P < 0.05, **P < 0.01. PTE: Pterostilbene; OGD/R: Oxygen-glucose deprivation/re-oxygenation; MDA: Malondialdehyde; GSH-Px: Glutathione peroxidase; ROS: Reactive oxygen species.

Excessive oxidative stress during OGD/R may promote cell damage. MDA and GSH-Px are commonly used indicators to reflect oxidative stress, so we tested the content of HT22 cells treated with different treatments by kits. MDA in the OGD/R group was markedly expanded, and GSH-Px was markedly decreased. After PTE treatment, this phenomenon was effectively reversed (Figure 2A and 2B). Next, we detected the level of oxidation in the cells. Flow cytometry, DHE and MitoSox were used to test the ROS intensity, superoxide anion and mitochondrial superoxide anion levels in the cells, respectively. The ROS intensity and the level of superoxide anion in the cells and mitochondria were significantly increased after OGD/R induction (Figure 2C–2F), and decreased significantly after PTE treatment. Combined with the above results, PTE can reduce the oxidation reaction in HT22 cells, reduce ROS accumulation, and increase the activity of antioxidant enzymes.

### PTE attenuates OGD/R-induced ferroptosis

**Figure 3.**
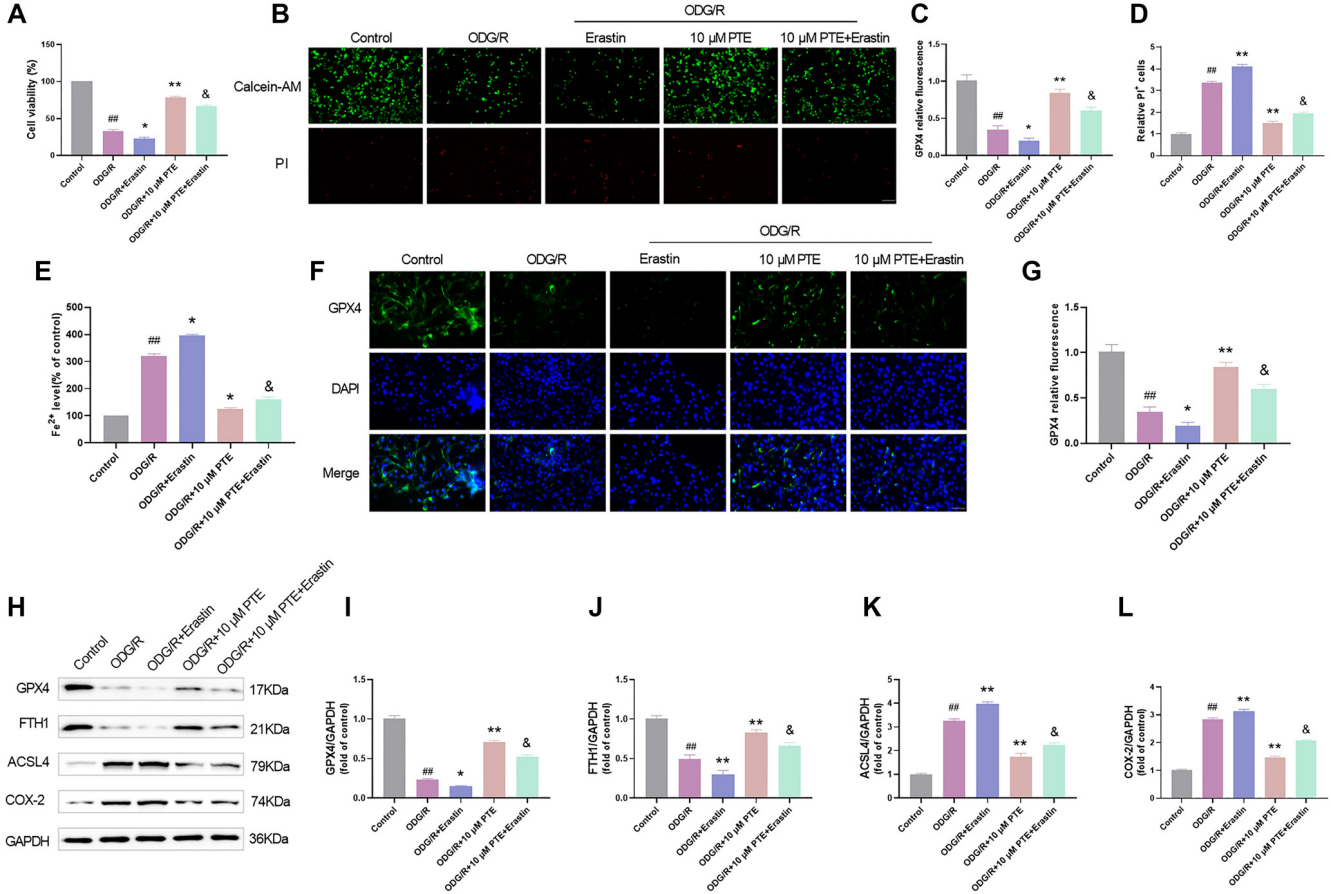
**PTE attenuates OGD/R-induced ferroptosis.** (A) HT22 cells were subjected to OGD/R modeling, and treated with 10 µM ferroptosis activator Erastin and 10 µM PTE 1 h before modeling. The cell viability was tested by MTT assay, and Erastin notably reduced the cell viability, but on this basis, the cell viability was notably elevated after PTE treatment; (B–D) Calcein-AM/PI detection of live/dead cells showed that Erastin notably diminished live cell count and expanded dead cell count, but it was significantly reversed after PTE treatment; (E) The content of Fe^2+^ was tested by the kit, and it was found that Erastin markedly grew the content of Fe^2+^, but it was notably reversed after PTE treatment; (F and G) Immunofluorescence detection of GPX4 showed that Erastin significantly reduced GPX4 level, but it was notably reversed after PTE treatment; (H and L) Ferroptosis-related proteins was tested by Western blot, Erastin significantly reduced GPX4 and FTH1 protein levels, increased COX-2 and ACSL4 protein levels, but on this basis, PTE treatment was significantly reversed. Compared with the Control group, ^##^*P* < 0.01; compared with OGD/R group, ^*^P < 0.05, **P < 0.01; compared with OGD/R+10 µM PTE group, ^&^*P* < 0.05. PTE: Pterostilbene; OGD/R: Oxygen-glucose deprivation/re-oxygenation; GPX4: Glutathione peroxidase 4; FTH1: Ferritin heavy chain 1; COX-2: Cyclooxygenase-2; ACSL4: Acyl-CoA synthetase long-chain family member 4.

Ferroptosis is a new mechanism of CIRI, which is closely associated with brain cell death, and an important feature of its occurrence is ROS production and lipid peroxidation. In view of the above results, the mechanism was vertified by the ferroptosis activator Erastin. The cell viability was significantly decreased after OGD/R treatment, the cell viability was significantly increased after treatment with PTE, but the cell viability was decreased after the application of Erastin (Figure 3A), suggesting that Erastin could weaken the effect of PTE, and PTE could affect the viability of OGD/R cells by interfering with ferroptosis. Erastin could further promote OGD/R cell death, while the effect of PTE group was opposite, but Erastin could weaken the inhibitory effect of PTE on OGD/R cell death (Figure 3B–3D), and the level of Fe^2+^ also illustrated this problem (Figure 3E), suggesting that activation of ferroptosis could enhance OGD/R-induced cell damage, and PTE could reduce CIRI cell damage by inhibiting ferroptosis.

GPX4 depletion is another important feature of ferroptosis [38], so we detected the fluorescence intensity of GPX4, which was notably diminished after OGD/R induction. Erastin could further promote GPX4 depletion, which could be significantly increased after PTE treatment. In addition, Erastin significantly weakened the effect of PTE (Figure 3F and 3G). In addition, FTH1, COX-2, and ACSL4 are also the star proteins of ferroptosis. We detected them by Western blot. The effects of Erastin and PTE on protein expression were consistent with the previous results (Figure 3H–3L). In summary, PTE alleviated Erastin-aggravated ferroptosis, demonstrating that PTE has a regulatory effect on iron metabolism in cell injury.

### PTE attenuates ferroptosis mediated by Wnt/β-catenin pathway

**Figure 4.**
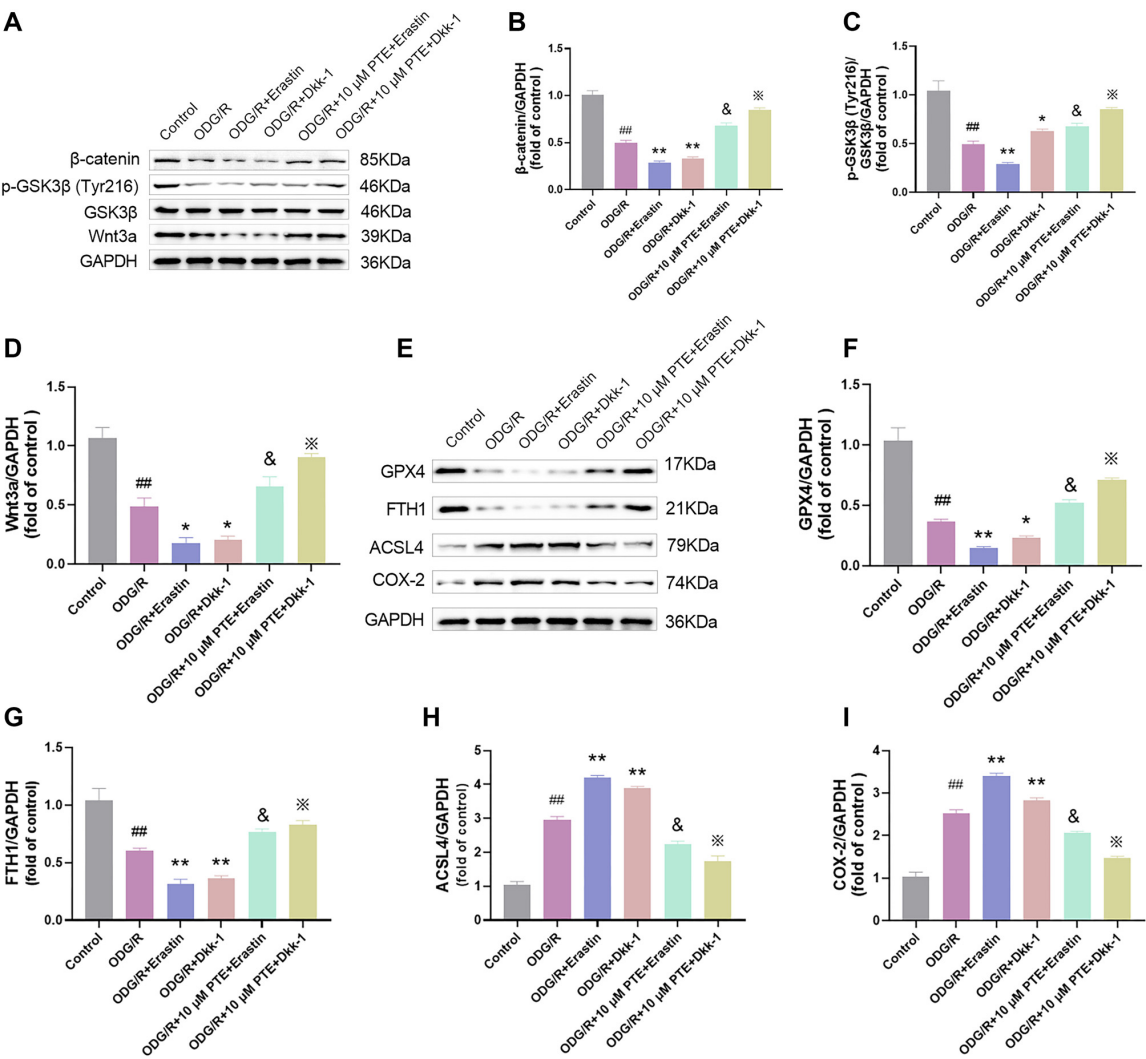
**PTE attenuates ferroptosis mediated by Wnt/β-catenin pathway.** (A–D) HT22 cells were exposed to modeling. One hour before modeling, 10 µM ferroptosis activator Erastin, 20 µg/mL Wnt signaling pathway inhibitor Dkk-1 and 10 µM PTE were used for treatment. Wnt/β-catenin pathway-related proteins were tested by Western blot. It can be seen that Erastin and Dkk-1 reduced β-catenin, p-GSK3β (Tyr216) and Wnt3a protein levels, but the protein levels were significantly increased after PTE treatment. (E–I) Ferroptosis-related proteins was tested by Western blot, Erastin and Dkk-1 significantly reduced GPX4 and FTH1 protein levels and increased COX-2 and ACSL4 protein levels, but on this basis, PTE treatment was significantly reversed. Compared with the Control group, ^##^*P* < 0.01; compared with OGD/R group, ^*^P < 0.05, **P < 0.01; compared with OGD/R+10 µM PTE group, ^&^*P* < 0.05; compared with OGD/R+10 µM PTE+Erastin group, ^X^*P* < 0.05. PTE: Pterostilbene; OGD/R: Oxygen-glucose deprivation/re-oxygenation; Wnt: Wingless-type MMTV integration site family member; GSK3β: Glycogen synthase kinase 3β; GPX4: Glutathione peroxidase 4; FTH1: Ferritin heavy chain 1; COX-2: Cyclooxygenase-2; ACSL4: Acyl-CoA synthetase long-chain family member 4.

Wnt/β-catenin pathway is strongly connected to ferroptosis [39], and it is involved in the pathophysiological process of cerebral ischemia injury [40]. So, whether PTE can inhibit ferroptosis through the Wnt/β-catenin pathway is worth exploration. We divided the experiment into control group, OGD/R group, OGD/R + Erastin group, OGD/R + Wnt signaling pathway inhibitor Dkk-1 (OGD/R + Dkk-1) group, OGD/R + 10 µM PTE + Erastin group and OGD/R + 10 µM PTE + Dkk-1 group, and then detected β-catenin, GSK3β and Wnt3a. The β-catenin, p-GSK3β (Tyr216) and Wnt3a protein levels were notably reduced after OGD/R treatment; Erastin and Dkk-1 can further aggravate the above situation, indicating that activation of ferroptosis could suppress the Wnt/β-catenin pathway; Likewise, the Wnt/β-catenin pathway was limited; PTE treatment can significantly increase its level and effectively alleviate the induction effect of OGD/R; when Erastin and Dkk-1 were added again, the effect of PTE was significantly reversed (Figure 4A–4D), indicating that PTE can reduce ferroptosis through the Wnt/β-catenin pathway. Next, we tested ferroptosis-related proteins, the expression changes of GPX4, FTH1, COX-2, and ACSL4 were consistent with the previous results (Figure 4E–4I), which further indicated that PTE could reduce cell ferroptosis mediated by Wnt/β-catenin pathway.

### PTE can reduce the damage of cerebral infarction volume and neurological function after MCAO in C57/BL6 mice

**Figure 5.**
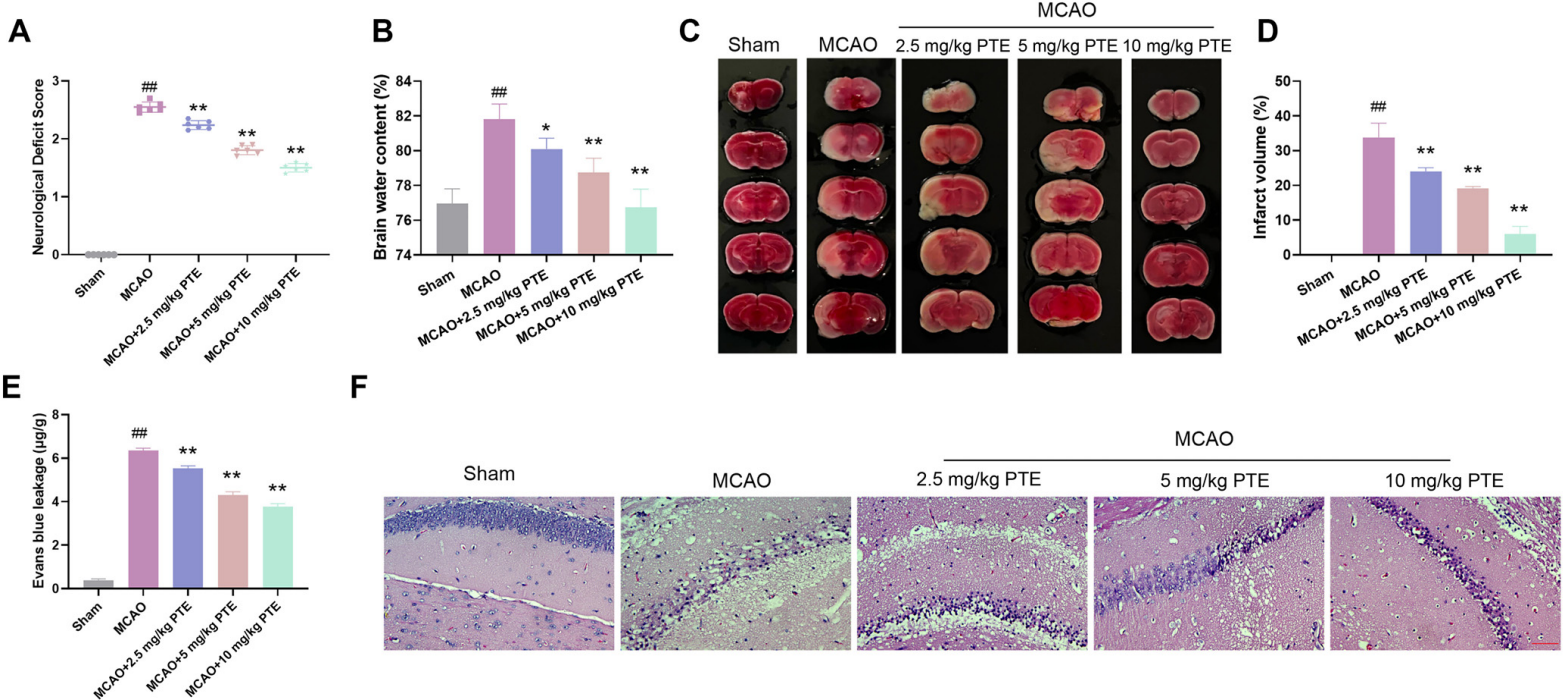
**PTE can reduce the damage of cerebral infarction volume and neurological function after MCAO in C57/BL6 mice.** (A) The neurological function was tested by Longa score method. The score of the MCAO model was notably elevated, and the neurological function score was obviously declined after PTE treatment; (B) The degree of brain edema was evaluated by brain wet/dry weight ratio. Severe brain edema was observed in the MCAO group, and brain edema degree was notably diminished after PTE treatment; (C and D) TTC staining of brain tissue sections showed that severe cerebral infarction occurred after MCAO treatment, and the degree of cerebral infarction was notably diminished after PTE treatment; (E) Evans blue leakage showed that the blood–brain barrier after MCAO treatment was seriously damaged, and obviously improved after PTE treatment; (F) HE staining of brain tissue sections showed that the cells after MCAO treatment were sparse and loose, forming voids, and the cell structure was severely broken. PTE could significantly improve the damage of brain tissue cell structure caused by CIRI and improve cell arrangement (×400, 50 µm). Compared with the Sham group, ^##^*P* < 0.01; compared with MCAO group, ^*^P < 0.05, **P < 0.01. PTE: Pterostilbene; MCAO: Middle cerebral artery occlusion; HE: Hematoxylin-eosin; TTC: Triphenyltetrazolium chloride; CIRI: Cerebral ischemia-reperfusion injury.

In view of the function and related mechanisms of PTE in vitro, we further explored whether PTE could affect the development of CIR in vivo. We used MCAO as a model and treated with 2.5, 5, and 10 mg/kg PTE. The neurological function score was markedly higher after MCAO treatment (Figure 5A), indicating that the model had obvious damage to the neurological function of the mice. The score was obviously reduced after PTE treatment, indicating that PTE can improve the damaged neurological function of mice. The brain wet/dry weight ratio can evaluate brain edema. The brain tissue of the MCAO group was severely edematous. After PTE treatment, brain edema degree was significantly improved (Figure 5B). Then the cerebral infarction was evaluated by TCC staining. There was no infarct area in the Sham group, while the MCAO group had obvious cerebral infarction, the volume increased significantly. After PTE treatment, the volume decreased notably (Figure 5C and 5D). The blood–brain barrier integrity was tested by evansblue leakage. The blood–brain barrier was significantly damaged after MCAO induction, and restored after PTE treatment (Figure 5E). HE staining was used to observe hippocampal CA1 region. In the MCAO group, the cells were arranged sparsely and loosely, forming voids, and the cell structure was severely broken. PTE could notably improve the damage of brain tissue cell structure caused by CIRI and improve cell arrangement (Figure 5F). In conclusion, PTE can protect the brain tissue of CIRI mice.

### PTE can reduce neuronal cell damage in hippocampal CA1 region of CIRI mice

**Figure 6.**
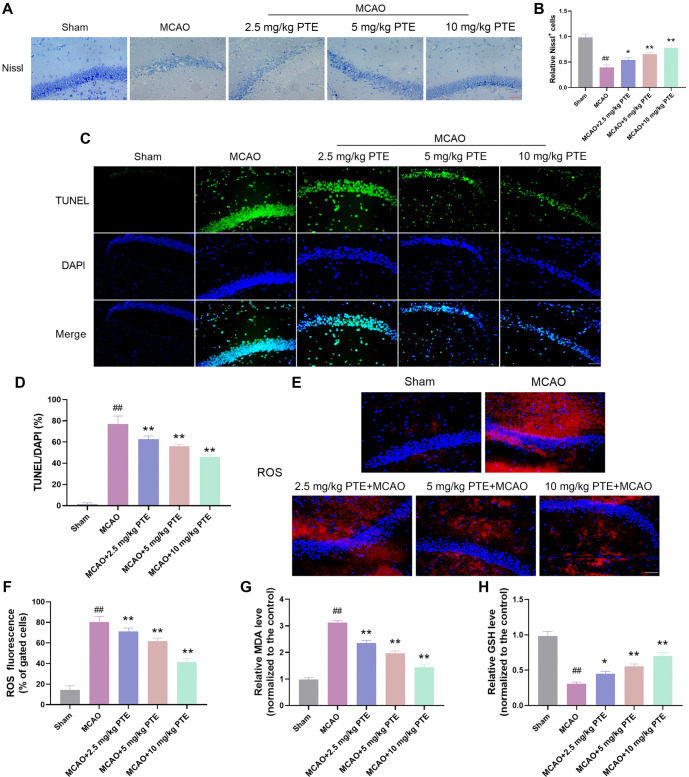
**PTE can reduce neuronal cell damage in hippocampal CA1 region of CIRI mice.** (A and B) Nissl staining of brain tissue sections revealed that the quantity of Nissl bodies after MCAO treatment was significantly lowered and significantly increased after PTE treatment (×400, 50 µm); (C and D) TUNEL staining revealed that the apoptosis of hippocampal CA1 cells after MCAO treatment was markedly increased, which was significantly decreased after PTE treatment (×400, 50 µm); (E and F) ROS was tested by flow cytometry, which showed that ROS after MCAO treatment was notably elevated and markedly decreased after PTE treatment (×400, 50 µm); (G and H) The content of MDA and GSH-Px was tested by kit. The content of MDA in MCAO group was notably elevated, and GSH-Px was obviously declined, which was obviously reversed after PTE treatment. Compared with the Sham group, ^##^*P* < 0.01; compared with MCAO group, ^*^P < 0.05, ^**^P < 0.01. PTE: Pterostilbene; MCAO: Middle cerebral artery occlusion; ROS: Reactive oxygen species; MDA: Malondialdehyde; GSH-Px: Glutathione peroxidase; CIRI: Cerebral ischemia-reperfusion injury.

Since PTE could improve the damaged neurological function of mice, we further explored this. Nissl bodies are often used as morphological indicators of neuronal survival [41]. Nissl body count in the hippocampal CA1 region of the Sham group was abundant, and the dendritic structure was normal. Nissl body count in MCAO group shrank significantly, and the dendritic structure disappeared. After PTE treatment, Nissl body count in mice elevated markedly and the dendritic structure recovered (Figure 6A and 6B). Then the apoptosis of hippocampal CA1 cells was tested by TUNEL fluorescence. The apoptosis of MCAO group was grew markedly, but after PTE treatment, the apoptosis was markedly diminished (Figure 6C and 6D). Subsequently, the oxidative stress in the CA1 region was detected. The ROS was increased significantly after MCAO treatment (Figure 6E and 6F), the MDA content also increased significantly (Figure 6G), and the GSH-Px content decreased significantly (Figure 6H). However, after PTE treatment, the ROS and MDA content diminished markedly, and the GSH-Px content increased notably. In short, PTE can protect CIRI mouse neurons by reducing oxidative damage.

### PTE attenuates brain ferroptosis in CIRI mice

**Figure 7.**
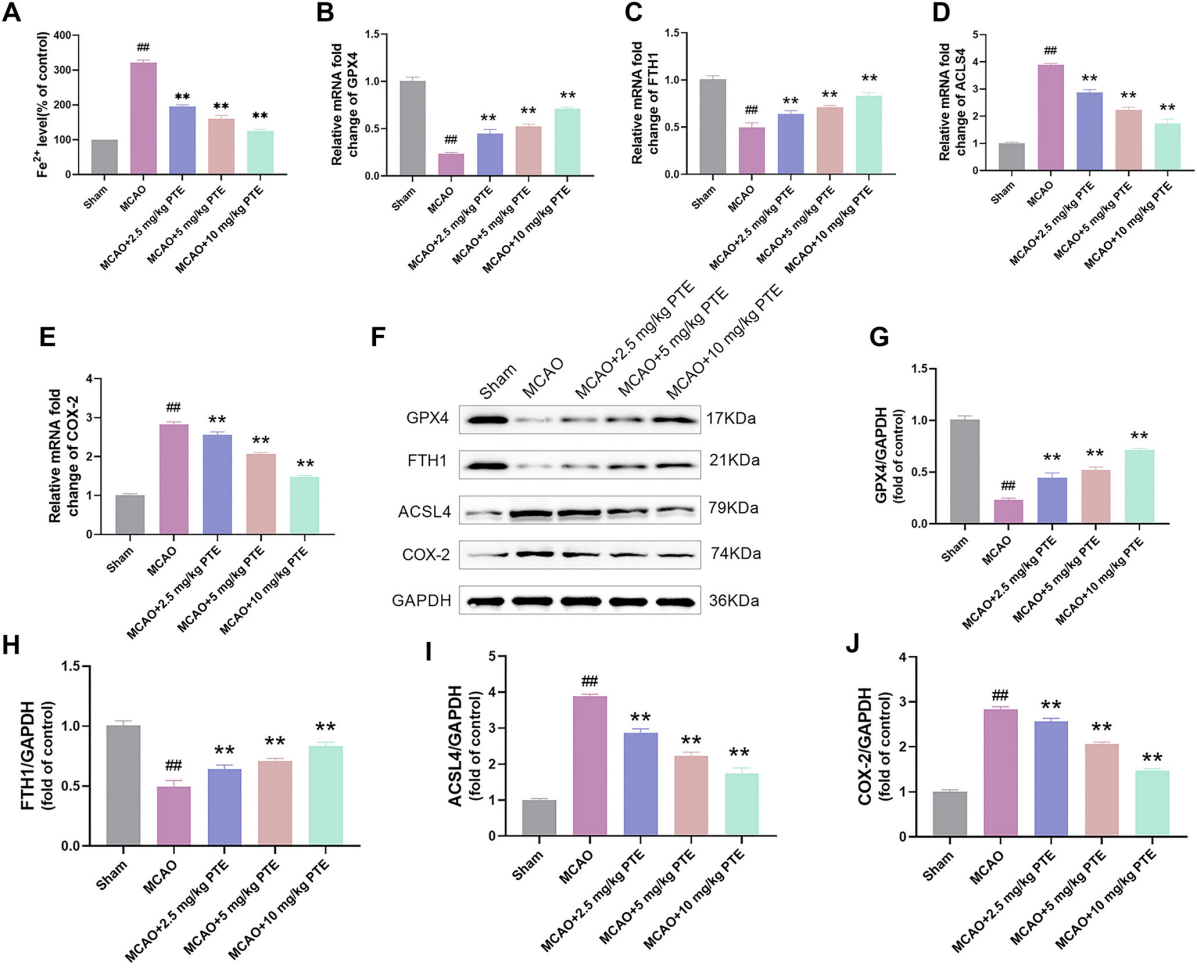
**PTE attenuates brain ferroptosis in CIRI mice.** (A) The content of Fe^2+^ was tested by the kit, and the content of Fe^2+^ in the MCAO group was notably raised, which was markedly lessened after PTE treatment; (B–E) The ferroptosis-related genes were detected by qRT-PCR. GPX4 and FTH1 mRNA levels in MCAO group were significantly decreased, while COX-2 and ACSL4 mRNA levels were significantly increased, which were significantly reversed after PTE treatment; (F–J) The ferroptosis-related proteins were tested by Western blot, GPX4 and FTH1 protein levels in the MCAO group were notably diminished, and COX-2 and ACSL4 protein levels were notably enhanced, which were significantly reversed after PTE treatment. Compared with the Sham group, ^##^*P* < 0.01; compared with MCAO group, ^*^P < 0.05, **P < 0.01. PTE: Pterostilbene; MCAO: Middle cerebral artery occlusion; GPX4: Glutathione peroxidase 4; FTH1: Ferritin heavy chain 1; COX-2: Cyclooxygenase-2; ACSL4: Acyl-CoA synthetase long-chain family member 4; CIRI: Cerebral ischemia-reperfusion injury.

Given that PTE can inhibit OGD/R-induced ferroptosis in vitro, we further explored whether PTE can inhibit ferroptosis in CIRI mice in vivo. Fe^2+^ content in the cerebral cortex was then detected using a kit. The Fe^2+^ content was rose considerably after MCAO treatment, and Fe^2+^ content was obviously decreased after PTE treatment (Figure 7A). Subsequently, we detected ferroptosis-related mRNA and protein. The GPX4 and FTH1 levels were notably reduced after MCAO treatment, and the COX-2 and ACSL4 levels were significantly increased. It was significantly reversed after PTE treatment (Figure 7B–7J). In summary, PTE can reduce ferroptosis in the brain tissue of CIRI mice. Combined with cell experiments, this study demonstrated that PTE can reduce CIRI by inhibiting ferroptosis.

### PTE can activate Wnt/β-catenin pathway in brain tissue of CIRI mice to reduce ferroptosis

**Figure 8.**
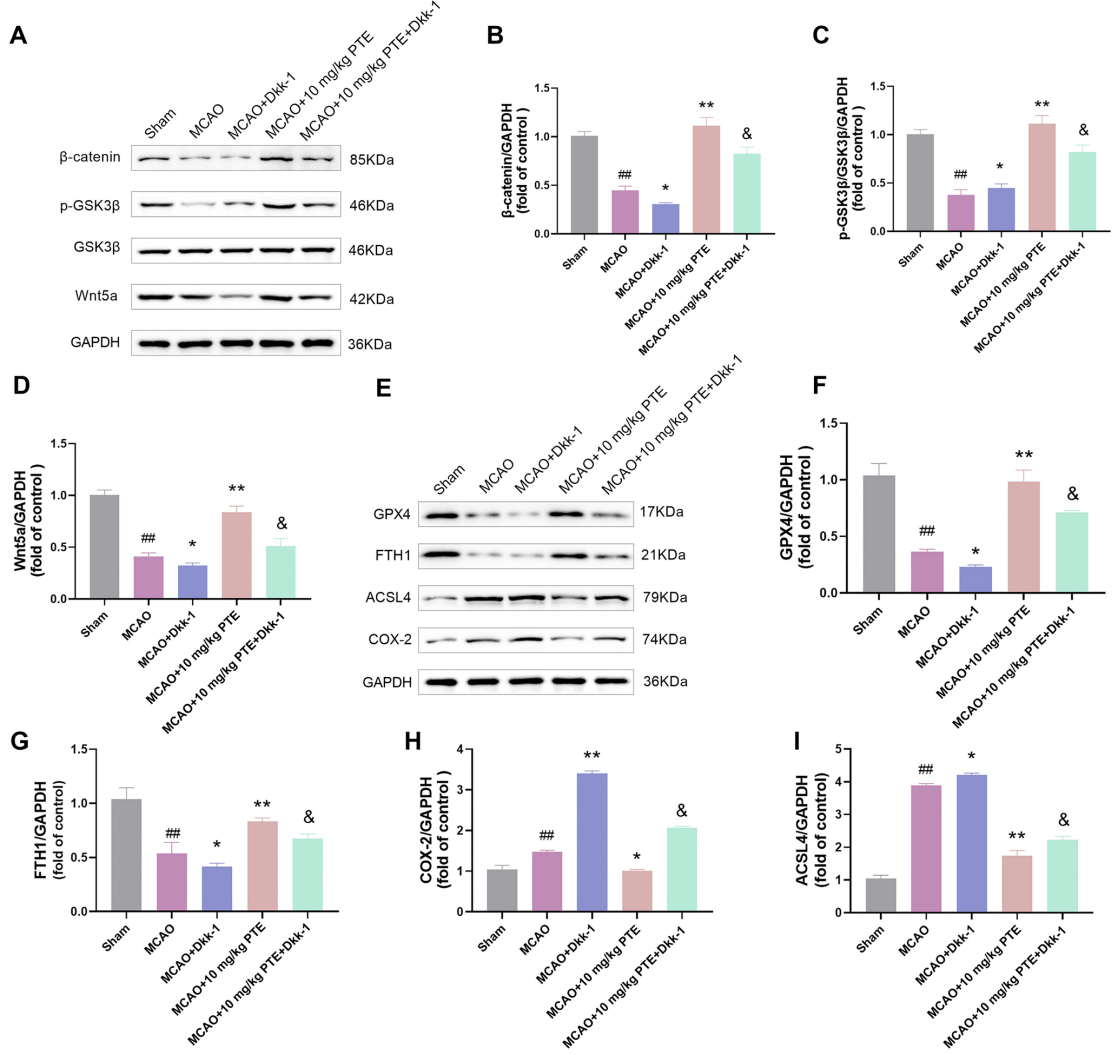
**PTE can activate Wnt/β-catenin pathway in brain tissue of CIRI mice to reduce ferroptosis.** (A–D) The mice were subjected to MCAO modeling, and treated with 20 µg/mL Wnt signaling pathway inhibitor Dkk-1 and 10 µM PTE. The Wnt/β-catenin pathway-related proteins were tested by Western blot. It can be seen that Dkk-1 reduces β-catenin, p-GSK3β (Tyr216) and Wnt3a protein levels, but on this basis, the protein level is significantly increased after PTE treatment; (E–I) Western blot analysis of ferroptosis-related proteins showed that Dkk-1 significantly reduced GPX4 and FTH1 protein levels, increased COX-2 and ACSL4 protein levels, but on this basis, PTE treatment was significantly reversed. Compared with the Sham group, ^##^*P* < 0.01; compared with MCAO group, ^*^P < 0.05, ^**^P < 0.01; compared with MCAO+10 µM PTE group, ^&^*P* < 0.05. PTE: Pterostilbene; MCAO: Middle cerebral artery occlusion; Wnt: Wingless-type MMTV integration site family member; GSK3β: Glycogen synthase kinase 3β; GPX4: Glutathione peroxidase 4; FTH1: Ferritin heavy chain 1; COX-2: Cyclooxygenase-2; ACSL4: Acyl-CoA synthetase long-chain family member 4; CIRI: Cerebral ischemia-reperfusion injury.

Since PTE can reduce ferroptosis through the Wnt/β-catenin pathway in vitro, we further explored in vivo. The mice were divided into Sham group, MCAO group, MCAO + Dkk-1 group, MCAO + 10mg/kg PTE group and MCAO + 10mg/kg PTE + Dkk-1 group. Then, the Wnt/β-catenin pathway protein and ferroptosis-related protein were tested. The change trend of protein expression was consistent with that in vitro (Figure 8A–8I), indicating that PTE could activate the Wnt/β-catenin pathway of CIRI mice to reduce ferroptosis. Combined with cell experiments, this study demonstrated that PTE can activate the Wnt/β-catenin pathway to prevent ferroptosis and reduce CIRI.

## Discussion

CIRI is a pathological injury caused by the re-recovery of blood perfusion after transient ischemia of brain tissue. The oxidative stress response triggered by CIRI can regulate the neuronal death–survival mechanism, cause irreversible damage to neurons, and cause apoptosis or death of brain cells [42, 43], destroy the blood–brain barrier, and therefore have a high risk of disability and death [44]. Nissl bodies are the characteristic structure of neurons and the main site of protein synthesis. When neurons are damaged, the number of Nissl bodies decreases, disintegrates or even disappears. During the process of injury repair, the number of Nissl bodies increases again, so Nissl bodies are regarded as indicator of neuronal survival [41]. In the cell experiment, we found that the cell damage was markedly expanded, death occurred. In animal experiments, the brain tissue damage of MCAO model mice is obvious [45, 46]. Insufficient blood supply after cerebral ischemic perfusion will damage brain tissue and cause brain edema, which will lead to neurological dysfunction in mice. In severe cases, there will be inability to walk independently and coma, neurological function score will be significantly increased, cerebral infarction volume will be expanded, and blood–brain barrier will be destroyed. Pathological staining showed that the morphology and structure of brain tissue cells were seriously damaged, and the staining of Nissl bodies in mouse brain tissue became lighter and the number decreased. After treatment with PTE, the above indicators were significantly improved, cell damage was significantly diminished, the cell viability was obviously elevated, cerebral infarction volume in mice was diminished, the blood–brain barrier was restored, and neuronal cells was improved, indicating that PTE can improve CIRI-induced neuronal damage. Although the 24-h time point is usually used for the MCAO model, we will extend the observation to multiple time points in subsequent studies to clarify whether the benefits of PTE are sustained, transient, or perhaps even amplified over time.

CIRI is closely connected to ferroptosis-related signals, including lipid peroxidation and high intracellular iron content [47, 48]. Free radicals are created when the body undergoes an oxidative reaction, which can result in cell membrane injury due to the peroxidation of unsaturated fatty acids in the phospholipids forming the membrane [49]. During cerebral ischemia, the production of free radicals leads to diminished antioxidant enzyme GSH-Px activity and heightened ROS and MDA content. When the endogenous mechanism of scavenging ROS after ischemia-reperfusion is severely damaged, it cannot effectively remove ROS, facilitating the formation of lipid peroxides, causing cell death [50]. Therefore, ROS, MDA content and GSH-Px activity can reflect the severity of the oxidation reaction. In this study, ROS, superoxide anion and MDA were obviously escalated in cell and animal experiments, GSH-Px was markedly diminished. After treatment with PTE, the above indicators were significantly improved, suggesting that PTE could inhibit the oxidation reaction of CIRI, reduce ROS accumulation, increase antioxidant enzymes activity, and reduce the level of lipid peroxidation.

Ferroptosis is a new mechanism of CIRI, which is closely associated with brain cell death. Inhibition of ferroptosis has gradually become an important method to protect brain cells. In this regard, we verified by the ferroptosis activator Erastin, the cell viability was notably reduced, and cell death occurred, indicating that activation of ferroptosis could increase cell damage. On the contrary, inhibition of ferroptosis can reduce CIRI cell damage and protect brain cells. Iron storage, lipid peroxidation, and a deficit in lipophilic antioxidants are key determinants that induce ferroptosis. When iron accumulates in the body, the iron content of the unstable iron pool in the cell increases. These increased Fe^2+^ facilitates lipid peroxidation via the Fenton reaction, generating hydroxyl radicals and activating lipoxygenase pathways, resulting in heightened oxidative stress [51]. The mechanism of lipid peroxidation is mainly associated with GPX4. GPX4 is important in protecting cells from lipid peroxidation and inhibiting ferroptosis [52], and is a specific indicator of ferroptosis [53]. Targeted intervention of GPX4 expression can inhibit ferroptosis [38]; COX-2 is an important marker of lipid peroxidation [54]; Increased expression of ACSL4 in brain tissue can lead to increased oxidative stress, which leads to ferroptosis and neuroinflammation [55]; FTH1 is an important protein for iron storage, vital for maintaining cellular iron levels [56]. With the increase of Fe^2+^ level in ischemic brain cells, iron chelation can reduce ischemic brain injury [19]. Intracellular iron overload can induce ferroptosis. Under the catalysis of Fe^2+^, it can cause the accumulation of ROS on membrane lipids, cause intracellular redox imbalance, and lead to cell death [57]. In this study, the GPX4 and FTH1 protein levels were notably declined in cell and animal experiments, and the COX-2 and ACSL4 protein levels were notably elevated. Erastin can further promote protein depletion, and PTE treatment can significantly increase its level. In addition, Erastin significantly weakens the effect of PTE, indicating that PTE reduces the ferroptosis aggravated by Erastin, which proves that PTE has an inhibitory effect on ferroptosis. Combined with the oxidation reaction, PTE reduced the levels of iron accumulation and lipid peroxidation, indicating that PTE had the effect of reducing the level of ferroptosis in CIRI.

Wnt/β-catenin pathway is essential to ferroptosis. Wang et al. [39] showed that engagement of Wnt/β-catenin signaling diminishes lipid ROS generation and thereby suppresses ferroptosis. Yin et al. [58] have shown that circAFF1 regulates GSK3β-mediated Wnt/β-catenin pathway by targeting miR-140-5p, thereby enhancing neuronal ferroptosis induced by cerebral hemorrhage. Studies have found that Wnt ligand proteins are the initiators of the Wnt/β-catenin pathway [59]. In the Wnt/β-catenin pathway, there is no activity in the absence of Wnt ligands [60]. The destruction complex formed by GSK3β, Axin, etc. promotes the conversion of β-catenin and maintains Wnt signaling in a closed state. On the contrary, with the Wnt ligand present, the Wnt ligand binds to its homologous receptor, suppresses the destroying complex activity, and causes the β-catenin protein to be stably transferred to the nucleus to activate the transcription of the Wnt target gene [61]. Wnt3a and β-catenin are key indicators of Wnt signaling pathway. After cerebral ischemia, Wnt3a, as an important signal transduction activator in Wnt signaling pathway, can activate the downstream key factor β-catenin. Wnt/β-catenin pathway is also related to ischemia injury. Under the condition of acute CIR, the activation of Wnt/β-catenin pathway can save the blood–brain barrier damage and microvascular hemorrhage caused by ischemia [62]. GSK3β is a decisive molecule in the Wnt/β-catenin signaling pathway and negatively regulates the Wnt/β-catenin signaling pathway. Once activated, GSK3β can directly catalyze the phosphorylation of β-catenin, thereby enhancing the degradation of β-catenin through ubiquitination [63]. The catalytic activity of GSK3β in the degradation of β-catenin depends on its own phosphorylation state [64]. For example, although phosphorylation of GSK3β can occur at Ser9 and Tyr216, phosphorylation of Ser9 impairs the catalytic activity of GSK3β, while phosphorylation of Tyr216 promotes its catalytic activity [65]. Therefore, β-catenin, GSK3β signaling protein and ligand protein Wnt3a in the Wnt/β-catenin pathway were tested. β-catenin, p-GSK3β, and Wnt3a protein levels were notably decreased in cell and animal experiments. Erastin and Dkk-1 could further aggravate the above situation. PTE treatment could significantly increase its level and effectively alleviate the induction effect of OGD/R. When Erastin and Dkk-1 were added again, the effect of PTE was significantly reversed. Our data indicate that the neuroprotective effect of PTE was closely associated with Wnt/β-catenin activity. Treatment with Dkk-1 inhibited the protective effect of PTE, suggesting that at least part of the mechanism of PTE involves Wnt/β-catenin pathway–mediated ferroptosis inhibition. To summarize, PTE could treat CIRI, which mechanism might be achieved by regulating the level of ferroptosis through Wnt/β-catenin pathway.

In the future, we will further clarify whether activation of the Wnt/β-catenin pathway is essential for PTE-mediated neuroprotection through other experiments (such as β-catenin knockdown, overexpression, or reporter assays). Similarly, combined with known a ferroptosis inhibitor (Ferrostatin-1), it will be confirmed whether the effect of PTE is specifically dependent on ferroptosis or is also affected by other antioxidant pathways.

## Conclusion

This article reveals the mechanism of PTE in improving CIRI. PTE can improve cerebral infarction, alleviate the pathological damage of cerebral cortex cells, reduce nerve damage, regulate the pathways associated with ferroptosis (iron metabolism, lipid peroxidation), and affect Wnt/β-catenin pathway. Overall, PTE alleviates CIRI in mice and HT22 cells, coincident with reduced ferroptosis and apparent activation of the Wnt/β-catenin pathway. It is confirmed that PTE partially mediates its neuroprotective effects through ferroptosis inhibition, likely in conjunction with Wnt/β-catenin signaling. Future studies should further confirm the causal role of the Wnt/β-catenin pathway and ferroptosis in PTE’s therapeutic potential. This research offers novel strategies for the treatment of CIRI with PTE, and provides a reliable reference for the targeted therapy of CIRI. Nevertheless, there are still some shortcomings in this research, and the pathogenesis of CIRI is very complicated. PTE improving CIRI may involve other pathways (Nrf2/HO-1), which need to be further explored in the future. In addition, the safety and efficacy of PTE in clinical applications need to be further evaluated in the future to provide a basis for the clinical application of PTE.

## Data Availability

The data supporting the findings of this study can be obtained from the corresponding author, upon request.
